# MRTF may be the missing link in a multiscale mechanobiology approach toward macrophage dysfunction in space

**DOI:** 10.3389/fcell.2022.997365

**Published:** 2022-09-12

**Authors:** Rocky An

**Affiliations:** ^1^ Department of Biological and Environmental Engineering, Cornell University, Ithaca, NY, United States; ^2^ Sibley School of Mechanical and Aerospace Engineering, Cornell University, Ithaca, NY, United States

**Keywords:** mechanobiology, microgravity, macrophage, multiscale, MRTF, radiation

## Abstract

Macrophages exhibit impaired phagocytosis, adhesion, migration, and cytokine production in space, hindering their ability to elicit immune responses. Considering that the combined effect of spaceflight microgravity and radiation is multiscale and multifactorial in nature, it is expected that contradictory findings are common in the field. This theory paper reanalyzes research on the macrophage spaceflight response across multiple timescales from seconds to weeks, and spatial scales from the molecular, intracellular, extracellular, to the physiological. Key findings include time-dependence of both pro-inflammatory activation and integrin expression. Here, we introduce the time-dependent, intracellular localization of MRTF-A as a hypothetical confounder of macrophage activation. We discuss the mechanosensitive MRTF-A/SRF pathway dependence on the actin cytoskeleton/nucleoskeleton, microtubules, membrane mechanoreceptors, hypoxia, oxidative stress, and intracellular/extracellular crosstalk. By adopting a multiscale perspective, this paper provides the first mechanistic answer for a three-decade-old question regarding impaired cytokine secretion in microgravity—and strengthens the connection between the recent advances in mechanobiology, microgravity, and the spaceflight immune response. Finally, we hypothesize MRTF involvement and complications in treating spaceflight-induced cardiovascular, skeletal, and immune disease.

## 1 Introduction

Macrophages (Mϕ) are an immune cell type featuring phenotypic flexibility in either fighting infection or promoting healing. Mϕ sense inflammation, activate upon sustained signaling, migrate to inflamed tissue, and secrete signaling cytokines. In spaceflight however, the unloading of weight in Mϕ has been known for at least 3 decades to dysregulate cytokine secretion (Chapes et al., 1992). The involvement of the cytoskeleton was first proposed then, but the underlying mechanism has been an open question since. In recent years, advances in mechanoimmunology have established that myocardin-related transcription factor-A (MRTF-A) is a cytoskeletal mechanosensor expressly involved in Mϕ pro-inflammatory activation and cytokine secretion (Yu et al., 2014). Thus, we aim to introduce MRTF in the context of spaceflight by taking a multiscale approach to past research on Mϕ dysregulation and other diseases.

### 1.1 Multiscale approaches

Multiscale approaches in mechanobiology consider molecules, single cells, tissues, and organs, including each of their varied responses across time scales, to resolve complex interactions between biology and mechanics (Mak et al., 2015; Fritzsche, 2020). Similarly complex, the combined environmental effect of spaceflight microgravity (apparent 10^–4^ × g) and radiation has been given a multiscale mechanobiology approach for cardiovascular disease (Basirun et al., 2021) and muscle/bone loss (Deymier et al., 2020), but not for immune dysregulation. Yet current immune studies in microgravity vary in scale from drop-towers (seconds) to ballistic flights (minutes) to long-term spaceflight (months), reviewed in detail by ElGindi et al. (2021), or microgravity is simulated for a few days in 3D random positioning machines (3D-RPM) and rotating wall vessel bioreactors (RWV), where constant rotation time-averages the gravity vector to be negligible (Hammond and Hammond, 2001).

Mϕ are commonly given multifactorial analysis (Cess and Finley, 2020; Orsini et al., 2021) because their phenotype is affected by a dynamic balance of extracellular cytokine signaling, intracellular crosstalk, immune cell-cell interaction, and mechanical and physiological environment (Finch-Edmondson and Sudol, 2016; Decano and Aikawa, 2018). These factors are space- and time-dependent, and thus differential changes observed across experimental timescales were often interpreted as an adaptation to microgravity (Meloni et al., 2006; Paulsen et al., 2015; Ludtka et al., 2021b). Instead of such broad interpretations, however, mechanistic understandings are necessary for safe, effective treatment of spaceflight diseases such as immune dysregulation (Crucian et al., 2018), cancer progression (Kim et al., 2021), circadian rhythm disruption (Simmet et al., 2013), and accelerated atherosclerosis (Meerman et al., 2021). For example, blood-circulating monocytes are recruited as pro-inflammatory Mϕ toward atherosclerotic lesions because of many factors including radiation (Patel, 2020), reactive oxygen species (ROS) (Wang Y. et al., 2014), adhesion proteins (Yang et al., 2005), and motility (Mukherjee et al., 2022)—all of which are afflicted by spaceflight.

**FIGURE 1:**
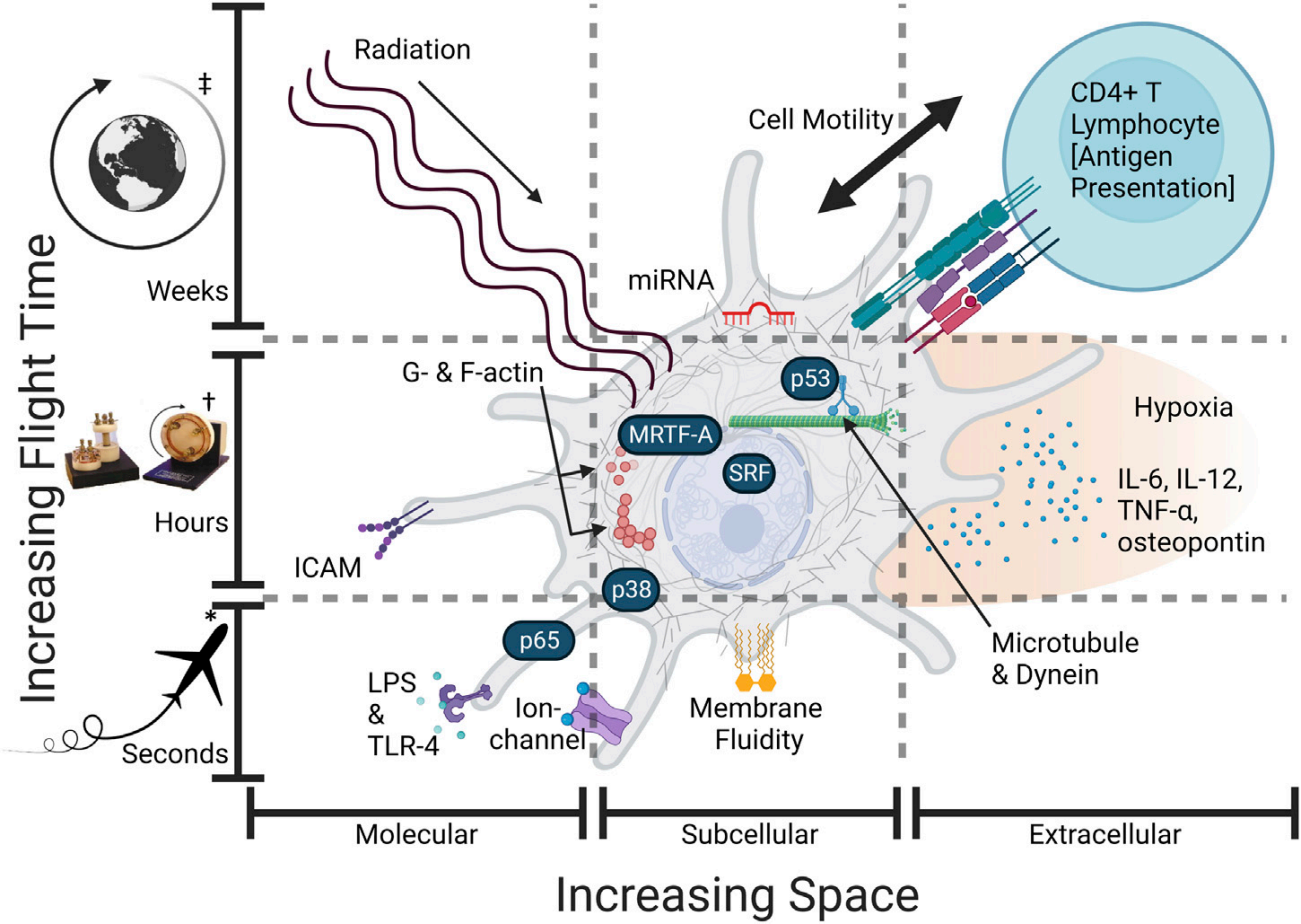
Some Mϕ spaceflight effects require more time or space. An overview of the altered spaceflight exposome (gravity, cytoskeleton, intracellular transport, hypoxia, radiation, intercellular signaling) and hypothetically relevant sensors and effector proteins: (LPS—lipopolysaccharide, TLR-4—t oll-like receptor 4, G-actin—globular actin, F-actin—filamentous actin, microtubules, dynein, p53—tumor protein P53, p38—mitogen-activated protein kinase p38, MRTF—myocardin-related transcription factor-A or megakaryoblastic leukemia 1 (*MKL1*), SRF—serum response factor, NF-κB/p65—nuclear factor kappa-light-chain-enhancer of activated B cells (p65 or RelA), IL-6—interleukin 6, IL-12, TNF-α—tumor necrosis factor-alpha, osteopontin, ICAM-1—intercellular adhesion molecule-1, miRNA—(microRNA such as miR-21—microRNA-21-5p). Spatial variation occurs across molecular, cellular, and physiological scales (increasing space). Time variation occurs from seconds in microgravity to months from long-term radiation (increasing time). Effects are not mutually exclusive and may interact at multiple scales, i.e., microgravity first acts alone and later acts in conjunction with radiation. Created with BioRender.com. *Parabolic Flight, †Simulated microgravity culture vessel (not flight), ‡Long-term orbital spaceflight.

Here, we apply a multiscale analysis in reviewing literature and data comparatively across spatial and temporal perspectives on microgravity, mechanotransduction, radiation, and crosstalk. First, we briefly describe individual spaceflight effects in increasing order of space and time (Figure 1). Then, we propose mechanisms for the most well-studied Mϕ phenotype changes in space: pro/anti-inflammatory activation, morphology, migration, and phagocytosis. To address knowledge gaps, we introduce the role of emerin—a putative gravi-sensitive nuclear envelope protein (Aventaggiato et al., 2020; Vahlensieck et al., 2022)—, novel microgravity mechanisms for arginase-1 (*ARG1*) regulation, and, most notably, a novel scale in the multiscale space milieu via the MRTF-A/SRF (serum response factor) pathway. Compared to live-cell imaging, transcriptomic analysis has traditionally been blind to the dynamic, intracellular localization of MRTF-A (Hipp et al., 2019; Kuchler et al., 2022). Furthermore, MRTF-A is currently not included in any KEGG database pathway, and its transcription program may be concealed by overarching pro-inflammatory signaling pathways. Mutations in MRTF cause severe immunodeficiency (Sprenkeler et al., 2021). Thus, introducing MRTF reinforces space studies that would otherwise have seemingly contradictory conclusions regarding suppression or activation of the pro-inflammatory (classical M1) response of the uniquely mechano-regulated Mϕ cell type.

### 1.2 MRTF-A transduces Mϕ pro-inflammatory signals

Mϕ pro-inflammatory activation and cytoskeletal reorganization occurs in a biphasic manner (Jain and Vogel, 2018; Ronzier et al., 2022): firstly in a chemical and secondly a mechanotransductive phase lasting 0–3 h and 3–24 h, respectively. In the first stage, activation of surface receptors induces NF-κB/p65 nuclear translocation. Secondly, actin polymerization modulates cytokine transcription/secretion via transport of MRTF-A to the nucleus where it slowly accumulates over 3 h and associates with serum response factor (SRF) or NF-κB/p65 transcription factors, or independently binds to SAP motifs of DNA (Olson and Nordheim, 2010; Gau and Roy, 2018; Zhou et al., 2021). The mechanosensitivity of MRTF-A is well-studied; if mechanical force induces polymerization of globular (G)-actin to filamentous (F)-actin, then G-actin-bound MRTF-A is released and translocated to the nucleus (simplified “classical” model):

F-actin ↔ G-actin ↔ MRTF-A←| Nuclear Envelope |→ SRF

**FIGURE 2:**
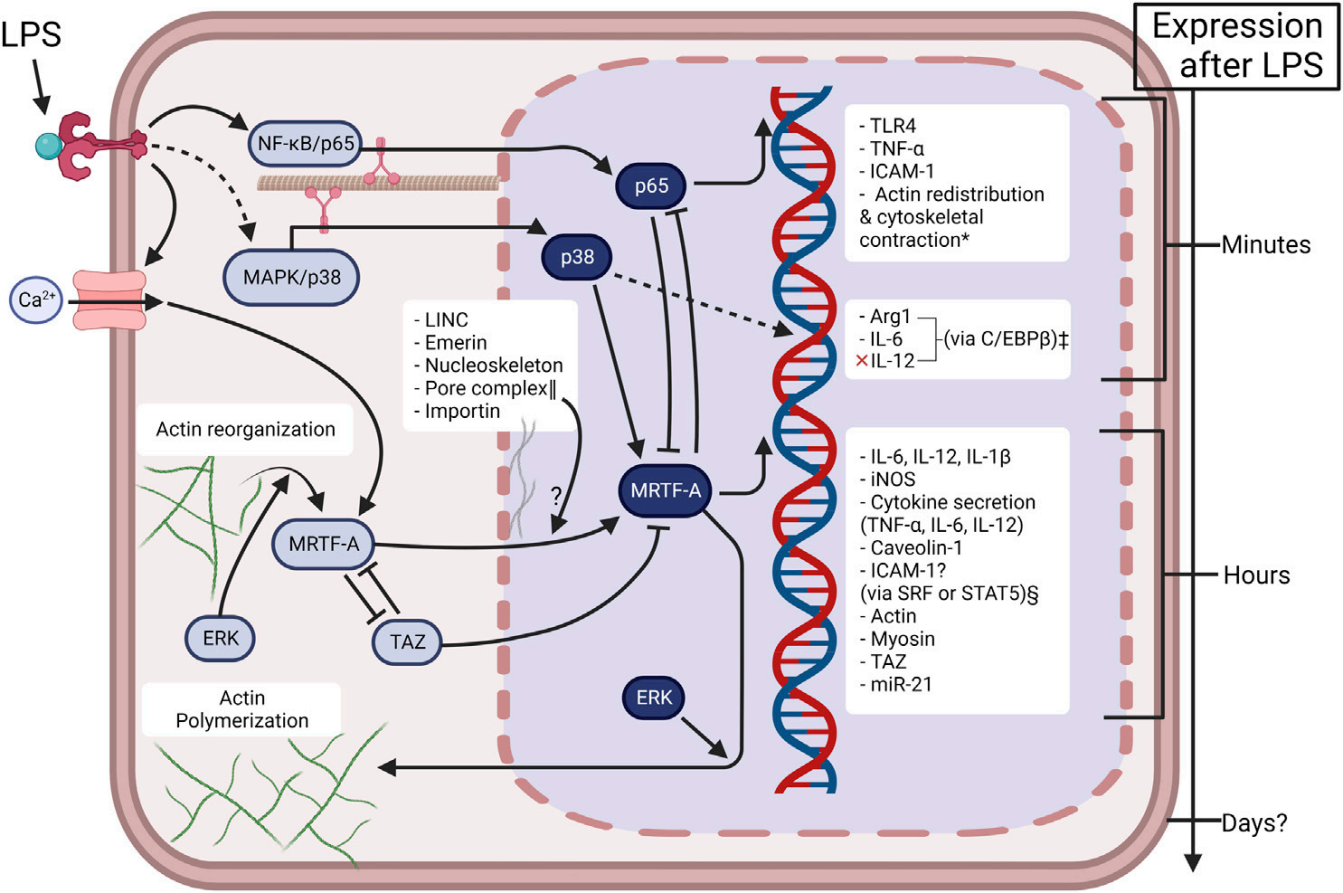
Mechanotransduction is inherently involved in Mϕ activation in a time-dependent manner. Under controlled conditions, LPS induces TLR4 “outside-in” signaling in the first few hours of activation. Sustained LPS induces “inside-out” signaling, resulting in activation of ion-channels and actin reorganization. This causes a second mechanotransductive phase via the accumulation of MRTF-A. The present figure depicts the current knowledge about MRTF in Mϕ activation, but many pathways are simplified for clarity. The complicated involvement of cellular mechanical mechanisms (LINC—linker of nucleoskeleton and cytoskeleton, emerin, ERK—extracellular signal-regulated kinase, YAP/TAZ—yes-associated protein/transcriptional coactivator with PDZ domain), or physical environments (substrate stiffness, spatial confinement, microgravity) alters the extent of Mϕ activation. The bidirectional interaction of p65 and MRTF does not fully inhibit them as some late-transcriptional genes are promoted by the p65 & MRTF complex e.g., *iNOS* (Miranda et al., 2021). Created with BioRender.com. *(Bian et al., 2017; Mu, 2018; Ronzier et al., 2022). ‡(Wang C. et al., 2015). §MRTF-A epigenetic regulation of ICAM-1 is likely dependent on cell type (Sullivan et al., 2011; Yu et al., 2014; Miranda et al., 2021) and is not known in Mϕ. ‖(Hoffman et al., 2020).

Figure 2 presents a simplified mechanistic overview of MRTF in Mϕ pro-inflammatory activation. Together with the comprehensive list of MRTF/SRF target genes by Esnault et al. (2014), inflammatory target genes include interleukin 6 (*IL6*), *IL1Β*, *IL12B,* and inducible nitric oxide synthase (*INOS* or *NOS2*) (An et al., 2017; Jain and Vogel, 2018; Yang et al., 2020). Other downstream effects include the secretion of pro-inflammatory cytokines IL-6, IL-12, and interestingly, tumor necrosis factor-α (TNF-α) (Jain and Vogel, 2018)—thus TNF-α secretion and *TNF-α* expression (p65 promoted) are regulated by specific mechanisms in Mϕ. This is supported with the understanding that Mϕ activation is metabolically regulated by epigenetic “brakes” (Ivashkiv, 2013), and that MRTF physically interacts with NF-κB/p65 resulting in the mutual inhibition of them both (Miranda et al., 2021) (Figure 2). Lastly, it is important to note that MRTF-A/SRF mediates actin and myosin gene expression (Guenther et al., 2019), thus facilitating “mechanoadaptation” (Dupont and Wickström, 2022). We interpret this delayed feedback loop for cytoskeletal remodeling as a possible mechanism for long-term adaptation to microgravity.

Many studies, reviewed by Sun et al. (2021), have found the Mϕ NF-κB inflammatory pathway to be unaffected by microgravity. If not caused by NF-κB/p65, then what is the mechanism of Mϕ phenotypic change? The microgravity effect on the MRTF-A/SRF pathway has not been explored in Mϕ and has been rarely explored in other cell types. Chang et al. (2012) analyzed astronaut T-cell transcriptomic profiles, finding the majority of downregulated genes to be promoted by SRF. Later, in a similar spaceflight study by Hughes-Fulford et al. (2015), it was found that microRNA-21 (miR-21) was downregulated. Relatedly, miR-21 is promoted by MRTF/SRF and is attributed to pro-inflammatory activation in Mϕ (Wang Z. et al., 2015; Li et al., 2017). We emphasize the importance of research in Mϕ because they are implicated in diseases associated with spaceflight, such as atherosclerosis—where plaque-associated Mϕ overexpress MRTF-A (An et al., 2019)—as well as circadian clock disruption (Shirato and Sato, 2022)—where Mϕ circadian clock components that regulate the timing of phagocytosis and motility are promoted by MRTF-A (Kitchen et al., 2020; Xiong et al., 2021).

## 2 Multiscale analysis in approx. increasing order of space and time

### 2.1 Microgravity-induced mechanical unloading

Mechanical factors such as shear stress, extracellular matrix (ECM)/tissue stiffness, and spatial confinement (Jain et al., 2019) correlate to immune regimes that govern Mϕ phenotype throughout the body. Innate immune system function necessitates Mϕ motility and phagocytosis, both of which require rapid cytoskeletal remodeling (Orsini et al., 2021). Likewise, microgravity—which in drop towers and parabolic flights is studied in second-long intervals—induces rapid cytoskeletal restructuring via actin depolymerization, but Mϕ repolymerize actin and correct it within minutes (Thiel et al., 2019). We speculate that feedback loops associated with the cellular level of actin polymerization are involved. For example, MRTF can recruit protein complexes associated with chromatin remodeling (Miranda et al., 2021). The actin nucleoskeleton also regulates and remodels chromatin (Venit et al., 2021), and is similarly restructured in microgravity resulting in the modulation of mechano-sensitive genes (Neelam et al., 2020).

Furthermore, the Mϕ cytoskeleton is physically linked with the cytoplasmic membrane. This linkage mediates motility and phagocytosis (Liu et al., 2020). Less studied in microgravity, there is evidence presented by Kohn et al. (2017) that microgravity increases lipid membrane fluidity or decreases membrane tension. If this is true, then lipid rafts could be disrupted, for instance allowing free diffusion of caveolin-1 (Le Roux et al., 2019)—a crucial protein for Mϕ phagocytosis (Li et al., 2005; Rubio et al., 2018). We mention that the quick response of the plasma membrane to mechanical forces may also play a role in the Mϕ oxidative burst reaction—which rapidly adapts to microgravity (Adrian et al., 2013; Thiel et al., 2017).

Membrane ion channels are also rapidly sensitive to membrane tension/fluidity and are known to have importance to inflammation, for instance inducing MRTF (Sharma et al., 2017). However, ion-channels are rarely studied in microgravity despite their mechanosensivity (Ludtka et al., 2021b). The two well-known mechano-sensitive Ca^2+^ ion-channels, transient receptor potential vanilloid 4 (TRPV4) and Piezo1, vary in activation responses to cytoskeletal structure, substrate stiffness/topology, and membrane tension/fluidity (Rahaman et al., 2014; Bryant et al., 2017; Botello-Smith et al., 2019; Romero et al., 2019; Sun et al., 2019; Krizaj et al., 2020; Orsini et al., 2021; Sianati et al., 2021). Another tension-sensitive ion channel, Hv1, is responsible for inducing superoxide production for the Mϕ oxidative burst reaction after phagocytosis (Ramsey et al., 2009). Interestingly, the channel has a mechanical history of up to 5 min (Pathak et al., 2016), which may have ramifications on microgravity platforms with cyclic loading e.g., 3D-RPM or RWV, or parabolic flight with a gravity period of ∼60 s.

### 2.2 Mechanotransduction

Gene expression is often studied on the timescale of hours in simulated microgravity bioreactors, which oscillate the gravity force usually between 10–15 rpm. Expression is not only induced by biochemical signaling, but also from the direct physical linkage of the cytoskeleton to the nucleoskeleton (Jaalouk and Lammerding, 2009). Remarkably, Guilluy et al. (2014) demonstrated nuclear stiffening under cyclic (0.14 Hz) mechanical force as small as 35 pN (near the weight of a Mϕ cell). They identified emerin, a ubiquitous nuclear lamin protein, to be involved independently from the nucleoskeleton. We identify emerin to be a potential confounding cause of nuclear stiffness discrepancies across simulated/spaceflight microgravity platforms—e.g., rotation frequency, substrate stiffness, or topology. Table 1 compares cell stiffness, migration, and filamentous actin (F-actin) levels across simulated microgravity platforms and culture methods that vary in substrate rigidity, adhesion, or extracellular matrix (ECM). Here, cells cultured on both rigid substrates and at 10–15 rpm (close to 0.14 Hz where emerin nuclear stiffening was observed) are more motile, stiffer, or exhibit greater actin polymerization (Janmaleki et al., 2016; Mao et al., 2016; Thompson et al., 2020; Wubshet et al., 2021), apparently contradicting general findings of spaceflight microgravity studies. It is worth noting that in normal gravity, cyclic tissue-stretching studies show significant MRTF translocation in fibroblasts at an optimum 0.1 Hz, but at relatively high levels of strain (Cui et al., 2015) compared to rotational simulated microgravity (1%–15% compared to almost 0%). Cytoskeletal strain may be negligible but emerin may not be. Emerin is known to be dependent on substrate-stiffness in modulating nuclear MRTF-A levels (Record et al., 2021).

**TABLE 1:** Simulated microgravity alters nuclear and cytoskeletal structural dynamics in various cell types and culture methods. Boldened results indicate concordance with observed spaceflight microgravity motility studies. Although in the field of cell adhesion and migration, the generalized effect of cell mechanical characteristics is still unclear (Mierke, 2021). The nucleus is the stiffest organelle and contributes the most to cellular stiffness (Qi et al., 2016). Increased actin polymerization generally increases nuclear size and stiffness *via* nucleoskeletal remodeling (Liu et al., 2019), thus reducing cellular motility (McGregor et al., 2016). Generally, cell motility is reduced in spaceflight and simulated microgravity across various cell types (Meloni et al., 2011).

Cell type	Platform	Culture method	Results	Study
J-111 monocyte	**3D-RPM, 60 rpm**	Chamber slides (Lab-Tek)	**↓ F-actin**	Meloni et al. (2006)
			**↓ Cell migration**	
Human breast epithelial cell	**3D-RPM, 2 rpm**	Cell culture flask (Fisher)	**↑ Nuclear volume**	Neelam et al. (2020)
MLO-Y4 Osteocyte	**RWV, 15 rpm**	Cell Rolling Tube (Thermo Scientific Forma^TM^)	**↑ Nuclear volume**	Yang et al. (2018)
			**↓ F-actin polymerization**	
Human umbilical vein endothelial cells	3D-RPM, ∼10 rpm	Petri Dish	↓ Cell stiffness	Janmaleki et al. (2016)
			**↓ F-actin, microtubules**	
Human osteoblast	3D-RPM, ∼10 rpm	Adherent cell culture	↓ Cell stiffness	Wubshet et al. (2021)
			**↓ F-actin**	
Rat bone marrow mesenchymal stem cell	RWV, 10 rpm	2D cell culture slide	**↑ Cell stiffness**	Mao et al. (2016)
			↑ F-actin polymerization	
Mouse mesenchymal stem cell	RWV, 15 rpm	SlideFlasks (2D plated cells)	↓ Nuclear stiffness (not significant)	Thompson et al. (2020)
			↓ F-actin (not significant)	

After a few minutes in microgravity, microtubule arrangement is disrupted (Papaseit et al., 2000) and in the span of 5 days, microtubules are shorter and wavier in Mϕ (Nabavi et al., 2011). Consequently, microtubule disruption induces the p38 mitogen-activated protein kinase (MAPK) pathway (Cuenda and Rousseau, 2007); thus we hypothesize that microtubule disruption is the cause of p38 MAPK induction, and further upregulation of *ARG1*, that is observed in Mϕ in simulated and spaceflight microgravity (Wang C. et al., 2015; Ludtka et al., 2021b). In fact, Mϕ *ARG1* expression is induced by perturbing microtubules via chemical methods, yet is not affected by chromatin remodeling nor by ECM stiffness (Meizlish, 2021). Alternatively, p38 MAPK induction is linked to mechanosensitive membrane proteins (Cuenda and Rousseau, 2007). The timescale difference between membrane proteins and microtubule arrangement could factor in Mϕ arginine level variation observed between short- and long-term spaceflight (Thiel et al., 2021).

### 2.3 Intracellular localization and transport

Upon sustained LPS stimulation, MRTF-A/SRF cytoskeletal mechanotransduction from Mϕ activation is a slow process that takes up to 4 h vs. a few minutes for the early stage of NF-κB (Bagaev et al., 2019). We hypothesize that delayed mechanotransduction causes experimentally observed “adaptations” to microgravity, and that inconsistencies observed across studies (Table 2) are time-dependent and pathway-specific. For example, cytokine expression/secretion of pro-inflammatory IL-6/IL-12/IL-1β is significantly downregulated after 4–24 h, concordant with our theory that actin disruption in microgravity inhibits the MRTF-A/SRF pathway. Interestingly, if normal gravity is restored post-48 h, then cytokine expression/secretion appears to recover (Table 2). Likewise, there is no time dependence of NF-κB-dependent TNF-α expression/secretion as it is consistently downregulated in both simulated and spaceflight microgravity. We also consider an alternative mechanotransductive pathway, p38 MAPK, in two studies where the data are available (Table 2), which may explain inconsistency in Table 2 regarding IL-6 and IL-12 expression/secretion, because p38 MAPK activation results in increased *IL-6* and decreased *IL-12b* expression (Wang C. et al., 2015).

**TABLE 2:** After Mϕ stimulation, cytokine responses are altered under microgravity over time. Boldened results indicate a reduction in pro-inflammatory cytokines TNF-α/IL-6/IL-12/IL-1β, and thus concordance with our theory of microgravity-based MRTF inhibition. Anti-inflammatory cytokines include IL-10. Protocols between studies varied the order between pro-inflammatory stimulation and microgravity.

Cell type	Platform	Culture method	Time after stimulation	Results	Study
U937 differentiated to Mϕ after RWV	RWV, 18 rpm	10-ml RCCS-D bulk vessels (Synthecon)	**1, 2, 3 h** after 12 h differentiation and 72 h RWV	**↓ IL-6 secretion, expression, exacerbated over time**	Wang et al. (2020)
				**↓ TNF-α secretion, exacerbated over time**	
				**↓ *TNF-α* expression**	
				↓ p38 MAPK pathway	
RAW 264.7 & primary mouse Mϕ	RWV, unspecified rpm	Adherent microcarrier beads	**4 h** after 24 h RWV	IL-1β expression (ns)	Wang C. et al. (2014)
				**↓ *TNF-α expression***	
				Unchanged MAPK pathway	
Primary mouse Mϕ	RWV, 12–25 rpm	Adherent microcarrier beads	**4** h after 24 h RWV	↑ IL-6 expression and concentration	Wang C. et al. (2015)
				↓ IL-12 subunit B expression	
				↑ p38 MAPK pathway	
			**24 h** after 24 h RWV	**↓ (less significant) IL-12 subunit B concentration**	
				↑ p38 MAPK pathway	
				**↓ *TNF-α expression***
RAW 264.7 murine Mϕ	RWV, 14 rpm	10-ml RCCS-D bulk vessels (Synthecon)	**48 h** after 48 h RWV	**↓ IL-6, IL-12 secretion**	Hsieh et al. (2005)
				**↓ TNF-α, NO secretion**	
Human blood monocyte stimulated with LPS	Spaceflight	*In vivo*, then whole blood cultured, and stimulated	under 1 g **48** **h,** after ∼350 h spaceflight	↓ IL-6 expression	Crucian et al. (2011)
				↑ IL-1β expression	
				**↓ *TNF-α* expression**	
				↓ IL-10 expression	
Mouse splenocyte stimulated with LPS	Spaceflight	*In vivo*, then flat-bottom plated, and stimulated	under 1 g **48** **h,** after ∼312 h spaceflight	↑ IL-6 secretion	Baqai et al. (2009)
				IL-12 (ns)	
				**↓ TNF-α secretion**	
				↑ IL-10 secretion	
RAW 264.7 murine Mϕ	RWV, 14 rpm	Adherent microcarrier beads	72 h RWV after **48 h** of stimulation	IL-6 (ns)	Ludtka et al. (2021a)
				↑ IL-12 secretion	
				**↓ TNF-α secretion**	
				↑ IL-10 secretion	

Our identification of MRTF-A/SRF pathway inhibition is the first time that altered Mϕ cytokine profiles have been linked to microgravity. Not only cytokines, but also a previous experiment (Hsieh et al., 2005) (Table 2) showed reduced nitric oxide (NO) secretion. In correlation, MRTF-A/SRF promotes iNOS (Yang et al., 2020) which is essential for killing pathogens after phagocytosis. We also conjecture that MRTF-A is a factor in impaired Mϕ phagocytosis in microgravity. MRTF-A-promoted genes involved in phagocytosis include caveolin-1 (*CAV1*) (Krawczyk et al., 2015) and intercellular adhesion molecule-1 (*ICAM-1*) (Zhong et al., 2021; Huang et al., 2022). Unfortunately, *ICAM-1* regulation by MRTF-A is not consistent across cell type and is unclear in Mϕ, and it may also be NF-κB-dependent (Fang et al., 2011; Hayashi et al., 2015). Additionally, the effect of microgravity on ICAM-1 regulation is controversial, varying between cell types (Paulsen et al., 2014; Tauber et al., 2017; Buravkova et al., 2018). For Mϕ, it is apparently time-dependent (Table 3), but no microgravity-linking mechanism has been identified yet.

**TABLE 3:** ICAM-1 surface expression over time in differentiated and non-differentiated Mϕ/monocytes. Simulated and spaceflight microgravity modulated U937 and human Mϕ ICAM-1 surface levels, but did not affect non-differentiated monocytes, even transcriptionally. Note, a microgravity phase of parabolic flight lasts 20 s, not enough time for differential transcription, thus differential surface expression of ICAM-1 may be attributed to membrane/cytoskeletal dynamics or other post-translational regulatory factors.

Cell type	Platform	Culture method	Time	Results	Study
Non-differentiated Monocytes, both stimulated and non-stimulated during flight					
U937 human monocyte	Parabolic flight	Nutrimix bag (B. Braun Melsungen)	20 s	No change in ICAM-1 surface expression	Paulsen et al. (2015)
U937 human monocyte	Sub-orbital rocket	Plastic Syringe	6 min	No change in ICAM-1 mRNA levels	Paulsen et al. (2015)
Differentiated Monocytes/Mϕ					
U937 human Mϕ-like monocyte	Parabolic flight	Nutrimix bag (B. Braun Melsungen)	20 s	**↑** Slight ICAM-1 surface expression	Paulsen et al. (2015)
Human primary Mϕ and U937 human Mϕ-like monocyte	RWV, 60 rpm	Serological pipette	24–120 h	**↑** Surface ICAM-1 **trending down (not significant) over time**	Paulsen et al. (2015)
U937 human Mϕ-like monocyte	Geocentric orbit	Polycarbonate slide	120 h	**↑** Surface ICAM-1	Paulsen et al. (2014)
				Severe disturbance of the cytoskeleton	
Primary human Mϕ	Low-earth orbit	Polycarbonate slide	264 h	↓ Surface ICAM-1	Tauber et al. (2017)
				No disturbance of the cytoskeleton	
			720 h	**↓↓** Surface ICAM-1	
				Altered cytoskeletal architecture	

ICAM-1 is a transmembrane protein found clustered in lipid rafts (Tilghman and Hoover, 2002) and anchored to the actin cytoskeleton (Schaefer et al., 2014). Induction of Mϕ ICAM-1 levels off after ∼12 h (according to Zhong et al. (2021) with 0, 12, and 24 h time points). Therefore, we postulate that MRTF-A is a delayed regulator of ICAM-1 expression in Mϕ. In a similar mechanism, Hayashi et al. (2015) found that in vascular endothelial cells, nuclear MRTF-A binds to NF-κB/p65, inhibiting p65 promotion of *ICAM-1*. The involvement of both NF-κB and MRTF-A/SRF pro-inflammatory pathways may explain the inconsistency across cell types about ICAM-1 expression in microgravity. For example in Table 3, we compare Mϕ to non-differentiated monocytes, a cell type that exhibits unchanged ICAM-1 levels during microgravity flights. Correspondingly, microarray analysis of these monocytes has shown that only two pathways are weakly altered after 6 min of pro-inflammatory stimulation: NF-κB, and the Epstein-Barr virus infection (Paulsen et al., 2015), which is related to the nuclear transport and function of p65 (Morrison and Kenney, 2004). These two pathways correlate with the first phase of pro-inflammatory activation. Comparatively in Mϕ and pre-differentiated monocytes, relative surface ICAM-1 levels trended downwards with time (Table 3). We interpret this as either as a resurgence of MRTF-A as the actin cytoskeleton recovers after 24 h or as a separate, unknown mechanism for *ICAM-1* downregulation in the long term. For example, microRNA-21 is downregulated in T-cells under microgravity (Hughes-Fulford et al., 2015). miR-21 is MRTF/SRF promoted, and attributed to “mechanical memory” of at least 20 days in bone mesenchymal stem cell (BMSC) fibrogenesis (Wang Z. et al., 2015; Li et al., 2017). Relatedly in Mϕ, miR-21 increases expression of ICAM-1 (Lu et al., 2020).

### 2.4 Hydromechanics of simulated and spaceflight microgravity

Altered hydromechanics: fluid shear against the walls of rotational culture vessels, gravitational buoyancy, buoyant mixing, and altered chemical/gas diffusion are commonly assumed to be negligible in simulated and spaceflight microgravity but are still part of the multiscale space milieu (Poon, 2020; An and Lee, 2022). For instance, Mϕ ROS production is quickly responsive to shear forces, which are observed in RPM bioreactors that rotate randomly (Brungs et al., 2019). Moreover, hydromechanical transport is a factor of altered phenotype of Mϕ when they are cultured on 2D vs. 3D substrate (Bhattacharya et al., 2020). Therefore, some microgravity hydrodynamic environments may exhibit altered chemical/gas diffusion, conferring local Mϕ hypoxia in culture. Overall effects may include activation of the p38 MAPK pathway (Paardekooper et al., 2018; Ke et al., 2019)—a pathway that exhibits contradictory activation or suppression in simulated microgravity (Table 2). Another potential effect is altered metabolism, as glycolytic lactic acid accumulation in culture may stimulate pro-inflammatory cytokine expression in Mϕ (Shi et al., 2021). Whether the microgravity altering effect on Mϕ metabolism can be attributed to both mechanical factors and hypoxic state remains to be elucidated.

Based on the paucity of evidence linking hypoxia with mechanotransduction, it is most likely there is only indirect interaction between the two. Independent of hypoxia, inflammatory cytokines such as IL-6, IL-18, and TNF-α induce hypoxia-inducible factors (HIF) in Mϕ (Vogel et al., 2019). HIF-1α is well studied in microgravity: Ludtka et al. (2021a) cultured Mϕ on adherent microbeads in RWV and observed no significant change of *HIF-1α* expression in Mϕ, yet observed upregulation of vascular endothelial growth factor (VEGF) secretion and downregulation of *TNF-α*. It is unclear whether this finding is caused by hypoxia, ROS, or mechanotransductive pathways. For example, the ERK/MAPK signaling pathway induces VEGF secretion across many cell types (Kim et al., 2009; Guo et al., 2020). Interestingly, myofibroblast differentiation is suppressed in hypoxia due to HIF-1α dependent inhibition of RhoA, a key remodeler of the actin cytoskeleton, overall hindering the MRTF/SRF pathway (Leinhos et al., 2019). Furthermore, hypoxia upregulates Mϕ expression of ICAM-1 likely in a p53 or NF-κB dependent manner (Gorgoulis et al., 2003). Generally, hypoxia polarizes Mϕ toward anti-inflammatory phenotypes (Ke et al., 2019). Thus, we hypothesize that hypoxia and microgravity act independently to suppress Mϕ pro-inflammatory phenotype.

### 2.5 Radiation and oxidative stress

The timespan of space radiation study ranges from weeks to months vs. microgravity study timespans of minutes to days. In contrast to hypoxia, we hypothesize that low-dose space radiation counteracts the effect of microgravity on Mϕ immune function. The immunomodulatory effect of radiation is dosage-dependent and depends on a multitude of factors including DNA damage, ROS generation, and modulation of inflammation pathways. A review in a cancer radiotherapy context by Wu et al. (2017) acknowledges that low-dosage radiation (comparable to spaceflight-relevant dosage) generally induces anti-inflammatory (alternative M2) activation—possibly by inactivation of p38 MAPK—but high doses induce pro-inflammatory (classical M1) activation, possibly by activation of p53—a well-studied transcription factor that stimulates DNA repair or apoptosis. Alternatively, p53 is transported by dynein on microtubules (Giannakakou et al., 2000), similar to p38 MAPK (see Section 2.2 *Mechanotransduction*).

The abrogation of Mϕ phenotypic disorder observed in space may be misattributed to adaptation to microgravity instead of the long-term effects of radiation. For instance, we hypothesize the apparent reversal of ARG1 (Thiel et al., 2021) and surface ICAM-1 expression between 11–30 days in orbital spaceflight (Table 3) to be caused by inactivation of either p38 MAPK or downregulation of miR-21 (see Section 2.3). A competing mechanism may be membrane-based: oxidative stress is caused by DNA damage and other radiation mechanisms e.g., upregulation of NADPH oxidase (NOX) causes ROS production (Sakai et al., 2018). ROS-based lipid peroxidation causes membrane fluidity reduction (de la Haba et al., 2013)—opposite to the effect of microgravity on fluidity (see Section 2.1). Nonetheless, there is evidence that space radiation alone is not significant for ROS production, but requires microgravity as a “synergistic potentiator” (Smith et al., 2012; Ran et al., 2016; Gomes et al., 2018). Considering the synergism between microgravity and radiation, it is possible that they involve MRTF-A and p65 (NF-κB), respectively; both transcription factors form a complex to promote *iNOS* (Miranda et al., 2021) and ROS-producing *NOX4* (Liu et al., 2018). Relatedly in vascular endothelial cells, oxidized low-density lipoprotein (oxLDL) causes cellular acetylation of MRTF-A promoting nuclear translocation and modulation of *ICAM-1* expression (Huang et al., 2022). Therefore, chronic ROS generation could be another mechanism for the apparent reversal of ICAM-1 surface expression in spaceflight.

### 2.6 Intercellular and physiological crosstalk

Mϕ dysregulation translates to impaired interaction with other immune cells. For example, T lymphocyte interaction is essential for antigen presentation, but may be slowed by Mϕ migration impairment in microgravity (Meloni et al., 2006). Additionally, Mϕ reduced surface ICAM-1 expression in spaceflight (Table 3) may hinder their adhesion and subsequent activation of CD4^+^ T lymphocytes (Lin et al., 2020). Not only considering immune cells, Han et al. (2022) observed the reduction of anti-inflammatory bacteria cultured under simulated microgravity. Wang et al. (2020) found live mouse hindlimb unloading (that is, a simulation of weightlessness by suspending hindlimbs in the air) to cause mouse gut microbiota dysbiosis and suppression of the p38 and ERK/MAPK pathways in intestinal Mϕ. Here, p38 and ERK was rescued by probiotics, thus microgravity may mechanically regulate the microbiota-immune axis. Zooming-out to the tissue scale, altered tensional homeostasis (such as that caused by microgravity mechanical unloading) impairs the transport of MRTF to the nucleus (McGee et al., 2011). Lastly, Mϕ are mediators of intercellular signals. As observed in coculture by Fu et al. (2019), radiation-induced apoptosis signaling is propagated by Mϕ, potentially increasing tissue damage. Damaged-cell intercellular signaling is enough to stimulate Mϕ differentiation/activation, regardless of Mϕ irradiation state.

Monocyte/Mϕ differentiation also depends on both microgravity and radiation. Shi et al. (2021) observed that microgravity suppresses differentiation of Mϕ to either pro-inflammatory or anti-inflammatory phenotype; yet, Coates et al. (2008) observed that radiation augments Mϕ differentiation. Earlier (Section 2.5), we have hypothesized that—regarding the innate immune response—radiation counteracts microgravity. But regarding bone degeneration, the effect of microgravity and radiation appears additive by increased fusion of monocyte/Mϕ in forming multinucleated osteoclasts (Bloomfield et al., 2016; Shanmugarajan et al., 2017). Osteal Mϕ also communicate locally with other cells: osteopontin, a versatile protein involved in bone cell migration, is promoted in osteoblasts under microgravity (Smith, 2020). Osteopontin also acts as a cytokine for Mϕ (Fantuzzi, 2003) generally promoting phagocytic activity (Schuch et al., 2016). Mϕ produces osteopontin when stimulated with anti-inflammatory IL-18 and IL-10 (Kobori et al., 2018), both of which are regulated by oxidative and mechanical stress. Thus, the effect of altered physical environments on Mϕ differentiation/activation may consequently dysregulate Mϕ chemical signaling to other tissues.

## 3 Conclusion and recommendations

In summary, we have discussed the hypothetical multiscale involvement of the MRTF-A/SRF pathway in the dysregulation of Mϕ under microgravity and radiation. MRTF-A is a regulator and adaptor of cytoskeletal architecture, migration, phagocytosis, ROS generation, cytokine secretion/expression, and adherence proteins. Thus, its involvement is a probable answer to the question of Mϕ phenotypic change in microgravity. However, MRTF-A/SRF has many complications; its function is dependent on cell type and is not completely understood in Mϕ (Liu et al., 2021). MRTF-A is post-translationally acetylated, phosphorylated, or SUMOylated by many factors, including intracellular crosstalk with other mechanotransductive pathways such as ERK (Panayiotou et al., 2016), YAP/TAZ (Lopez-Hernandez et al., 2021), and p38 MAPK in Mϕ (Ronkina et al., 2016), that alter its cellular localization. Crosstalk with MRTF is also bidirectional (Speight et al., 2016), so we suggest that MRTF is a culprit in impaired nuclear translocation of TAZ under simulated microgravity—as observed by Chen et al. (2016) to occur in BMSC in a noncanonical, F-actin-dependent manner. Furthermore, the nuclear transport of MRTF depends on nuclear lamina-associated proteins as well as cytoskeletal/nucleoskeletal architecture (Ho et al., 2013; Sidorenko et al., 2022). Related mechanical factors such as shear stress, vibration, and oscillation in simulated microgravity bioreactors may influence MRTF translocation. Not only mechanical but also chemical factors, such as hypoxia and oxidative stress, induce the MRTF/SRF pathway in Mϕ (Yang et al., 2020). Therefore, we recommend that future studies attempt to pinpoint MRTF-A/SRF modulation to one of these factors, not excluding microgravity.

We have primarily discussed the connection of MRTF-A to the actin cytoskeleton. However, we also recommend further study in microtubule disruption that may alter the p38 MAPK pathways. p38 MAPK is known to mediate MRTF-A phosphorylation, the consequence of which was found recently by Zhang M. et al. (2021) to be activation of the MRTF-A/p65 complex to promote *IL-6* in Mϕ. Furthermore, the consequence of radiation damage on microtubules is rarely studied although may be negligible (Zaremba and Irwin, 1981; Bruni et al., 2020). It is possible that radiation alters the transport of p38 MAPK and p65 NF-κB on microtubules. Thus, the two separate effects may modulate different pathways: NF-κB may depend on radiation and MRTF/SRF may depend on microgravity. To test this, we first recommend co-quantification of the MRTF-A vs. p65 NF-κB nuclear/cytoplasmic ratio, compared with the F/G actin ratio, under simulated microgravity followed by such in simulated radiation.

Mϕ are one of the most radioresistant and redox-resistant cell types, important for their role in the clearance of radiation-damaged, apoptotic cells (Meziani et al., 2018). However, Mϕ are mechano-sensitive and uniquely mechano-regulated (Sullivan et al., 2011) as described previously (Section 1.1). Importantly, the dominant effects of microgravity vs. radiation depend on cell type, thus directed treatment of spaceflight diseases should be specific to cell type. For example, spaceflight acceleration of atherosclerosis could be treated by activating p53, as it plays a crucial role in preventing the disease (Merched et al., 2003). However, p53 in Mϕ potentiates anti-inflammation and is already upregulated in microgravity (Shi et al., 2021), thus by activating p53 we may inadvertently expedite spaceflight immune dysregulation.

MRTF-A is widely expressed across many cell types and is implicated in cardiovascular, musculoskeletal, and immune diseases (Gau and Roy, 2018) relevant to spaceflight. For instance, MRTF-A is upregulated in blood-circulating Mϕ associated with atherosclerotic lesions, thus a drug that supplants MRTF-A (Velasquez et al., 2013; Yu-Wai-Man et al., 2017) may inadvertently accelerate atherosclerosis in space. Similar conclusions can be made with spaceflight diseases such as non-alcoholic fatty liver disease (Beheshti et al., 2019), related to MRTF (Zhang L. et al., 2021). Currently, no safe drugs have been proven for the treatment of space-induced cardiovascular disease, and evaluations of potential drugs is often contradictory (Meerman et al., 2021). In conclusion, future investigation of treatment for spaceflight diseases can be improved by a multiscale mechanobiological understanding of the consequence of microgravity × radiation environments on Mϕ. Our work contributes to this understanding by introducing MRTF.
